# The Benefit of Web- and Computer-Based Interventions for Stress: A Systematic Review and Meta-Analysis

**DOI:** 10.2196/jmir.5774

**Published:** 2017-02-17

**Authors:** Elena Heber, David Daniel Ebert, Dirk Lehr, Pim Cuijpers, Matthias Berking, Stephanie Nobis, Heleen Riper

**Affiliations:** ^1^ Department of Health Psychology and Applied Biological Psychology Institute of Psychology Leuphana University Lueneburg Lueneburg Germany; ^2^ Division of Online Health Training, Innovation Incubator Leuphana University Lueneburg Lueneburg Germany; ^3^ Faculty of Social and Human Sciences University of Southampton Southampton United Kingdom; ^4^ Department of Clinical Psychology and Psychotherapy Friedrich-Alexander-University Erlangen Nuremberg Erlangen Germany; ^5^ Department of Clinical, Neuro and Developmental Psychology Vrije Universiteit Amsterdam Amsterdam Netherlands; ^6^ Telepsychiatric Centre University of Southern Denmark Odense Denmark

**Keywords:** stress, mental health, internet-based interventions, review, randomized controlled trial, meta-analysis

## Abstract

**Background:**

Stress has been identified as one of the major public health issues in this century. New technologies offer opportunities to provide effective psychological interventions on a large scale.

**Objective:**

The aim of this study is to investigate the efficacy of Web- and computer-based stress-management interventions in adults relative to a control group.

**Methods:**

A meta-analysis was performed, including 26 comparisons (n=4226). Cohen *d* was calculated for the primary outcome level of stress to determine the difference between the intervention and control groups at posttest. Analyses of the effect on depression, anxiety, and stress in the following subgroups were also conducted: risk of bias, theoretical basis, guidance, and length of the intervention. Available follow-up data (1-3 months, 4-6 months) were assessed for the primary outcome stress.

**Results:**

The overall mean effect size for stress at posttest was Cohen *d*=0.43 (95% CI 0.31-0.54). Significant, small effects were found for depression (Cohen *d*=0.34, 95% CI 0.21-0.48) and anxiety (Cohen *d*=0.32, 95% CI 0.17-0.47). Subgroup analyses revealed that guided interventions (Cohen *d*=0.64, 95% CI 0.50-0.79) were more effective than unguided interventions (Cohen *d*=0.33, 95% CI 0.20-0.46; *P*=.002). With regard to the length of the intervention, short interventions (≤4 weeks) showed a small effect size (Cohen *d*=0.33, 95% CI 0.22-0.44) and medium-long interventions (5-8 weeks) were moderately effective (Cohen *d*=0.59; 95% CI 0.45-0.74), whereas long interventions (≥9 weeks) produced a nonsignificant effect (Cohen *d*=0.21, 95% CI –0.05 to 0.47; *P*=.006). In terms of treatment type, interventions based on cognitive behavioral therapy (CBT) and third-wave CBT (TWC) showed small-to-moderate effect sizes (CBT: Cohen *d*=0.40, 95% CI 0.19-0.61; TWC: Cohen *d*=0.53, 95% CI 0.35-0.71), and alternative interventions produced a small effect size (Cohen *d*=0.24, 95% CI 0.12-0.36; *P*=.03). Early evidence on follow-up data indicates that Web- and computer-based stress-management interventions can sustain their effects in terms of stress reduction in a small-to-moderate range up to 6 months.

**Conclusions:**

These results provide evidence that Web- and computer-based stress-management interventions can be effective and have the potential to reduce stress-related mental health problems on a large scale.

## Introduction

Chronic stress can lead to serious psychological and physical implications, such as depression [1], sleep problems [2], neck and shoulder disorders [3], and an increased risk of coronary heart disease events [4] and related mortality [5]. Substantial economic costs of stress and stress-related psychological disorders arise as a result of absenteeism, presenteeism, productivity loss, and high staff turnover [6]. Given that stress represents a major threat to public health, effective and scalable solutions to accommodate the demand for stress-management interventions are needed.

The evidence base on traditional face-to-face stress-management interventions is comprehensive, showing small-to-moderate overall effects in reducing adverse outcomes for mental health [7]. In their meta-analysis on interventions for work-related stress, van der Klink et al [8] reported a combined effect size of Cohen *d*=0.34 across all studies. The mean effect size was Cohen *d*=0.33 for depression and Cohen *d*=0.54 for anxiety for interventions that focused on individuals [8]. Richardson and Rothstein’s more recent meta-analysis [9] on occupational cognitive behavioral, relaxation, organizational, multimodal, or alternative stress-management interventions yielded somewhat larger effects, with an overall effect of Cohen *d*=0.53, an effect of Cohen *d*=0.68 for anxiety, and Cohen *d*=0.73 for stress. Evidence consistently shows cognitive behavioral interventions to be the most effective, with Cohen *d* values ranging from 0.68 [8] to 1.16 [9]. Some evidence also suggests that shorter interventions (1-4 weeks) are more effective than longer interventions; however, this effect may be confounded by treatment type [9].

A promising medium to facilitate the dissemination of effective mental health interventions is the Internet. In recent years, Web-based interventions for the prevention and treatment of a range of psychological conditions have proliferated [10-14]. Computer- and Web-based interventions are perceived to offer several advantages that may overcome some of the limitations of face-to-face approaches, including anonymity, 24/7 availability, reduced costs in terms of traveling to courses for both participants and instructors, high scalability, and a low access threshold. Enabling participants to be reached earlier than in classical face-to-face trainings, such interventions may have the potential to prevent the onset of more severe mental health problems [15-18]. Internet-based interventions have been found to be effective in clinical applications to address, for instance, depression [10], anxiety [19], and sleep disorders [20]. However, only a few randomized controlled trials (RCTs) have investigated Web-based stress-management interventions. Research on the eff0.19

Existing Web-based stress-management trainings differ in various aspects, such as the intervention content, length, and guidance, which may influence their efficacy. First, the theoretical basis of such interventions is diverse, including cognitive behavioral therapy (CBT) [21-26], third-wave cognitive behavioral interventions (TWC) [27-37], the use of olfactory care products [38], and physical exercise programs [39]. Second, the length and number of intervention sessions vary, ranging from short 2-week interventions [33] to interventions that allow access over several months [37]. Some interventions encourage participants to log on only as often as they like, with no specified sessions or requirements to complete the entire intervention [21], whereas others have fixed weekly appointments (eg, in an online virtual classroom) [37]. Third, the existing Web- and computer-based studies include both guided and unguided interventions and thus differ in the amount of human support given to participants during the intervention. In the guided format, individuals normally receive written feedback from a coach on the exercises that they complete within the training. In the study of Ruwaard et al [24], for example, clinical psychology students provided weekly feedback on exercises according to an instruction manual and reminded participants in cases of noncompletion. In contrast, Billings et al [21] used a less intensive, high-latitude format in which no feedback was provided.

In recent years, the number of studies on Web- and computer-based stress management has been rising. The overall effect of Web-based stress-management interventions and the influence of specific formats of treatment delivery remain unclear. Considering the demand for effective, scalable stress-management trainings and the enormous potential of Web- and computer-based interventions, there is a need to synthesize the results of existing studies. This meta-analysis aims to integrate the effects of Web-based stress-management interventions on the level of stress of adults. Additionally, effects on depression and anxiety will be assessed. The following research questions are addressed:

Are Web- and computer-based stress-management interventions effective in reducing stress, depression, and anxiety relative to a control group?

Are there differences in effect sizes concerning (a) the study quality, (b) the level of guidance, (c) the theoretical basis, and (d) the length of the intervention?

## Methods

### Eligibility Criteria

We considered RCTs from 1990 to May 2016 in which adult participants (older than 18 years) experienced stress and were participating in the trial to decrease their stress levels. The search was initially conducted in August 2013 and repeated in May 2016 to ensure it was as current as possible. Studies prior to 1990 were excluded; due to the rapidly changing technology in this field, these programs cannot be compared to the current interventions that are likely to be delivered to participants seeking help. The primary intervention target of included studies needed to be a reduction of stress within the target group. We excluded trials that targeted participants with medical conditions (eg, cancer, tinnitus, headache); caregivers of people with medical conditions (eg, caregivers for dementia); and participants with psychiatric disorders (eg, depression, anxiety or posttraumatic stress disorder), posttraumatic symptoms, postpartum emotional distress, or bereavement. The studies included needed to compare an intervention to any type of comparison group. There were no restrictions with regard to dosage or intensity, delivery, duration, frequency or timing of delivery, or the type of delivery channel (eg, email, Web-based, computerized). Trials investigating stress management as merely one part of the intervention (eg, alongside depression or anxiety) were included only if the primary goal of the intervention was to reduce stress. Furthermore, trials conducted in the context of the promotion of well-being rather than stress reduction were excluded. The studies had to report at least one instrument that claimed to measure stress levels. For the main analysis, we considered postintervention data. We also included follow-up data (when available) to assess longer-term effectiveness.

### Information Sources and Search Strategy

The search strategy for this meta-analysis was created with four categories of search terms. We defined the search terms to meet the following criteria: (1) stress reduction, (2) evaluation of an intervention program, (3) application of a RCT as an experimental design, and (4) delivery in a Web- or computer-based context. A detailed description of the search terms can be found in the Multimedia Appendix 1.

The specified search strategy was applied in three major database search engines (PsycINFO, PubMed, and Cochrane). Additionally, manual searches in key journals (eg, *International Journal of Stress Management*, *Journal of Medical Internet Research*, *Journal of Occupational Health*, *Scandinavian Journal of Work*, *Environment and Health*) and in the reference lists of the included studies were conducted.

### Study Selection

After removing duplicates of the articles identified, two researchers (EH and SN) independently screened all titles and abstracts based on the inclusion and exclusion criteria, and two researchers (EH and DL) assessed the full-text articles for eligibility. The researchers who assessed the relevance of the studies were not blind to the authors, institutions, journal of publication, or results.

### Data Extraction

Data were extracted concerning the origin, the number of participants, age, gender, comparison group, outcomes, theoretical basis, length, guidance, the risk of bias, and follow-up data. In cases of insufficient description, the primary investigators of the respective studies were contacted to obtain missing information.

### Risk of Bias

Two researchers (EH and DL) assessed the risk of bias of the included studies in accordance with the Cochrane Guidelines [40]. Thereby, sequence generation, allocation concealment, blinding, incomplete outcome data, and selective outcome reporting were judged.

### Power Calculation

Because we expected only a limited number of studies with relatively small sample sizes, we conducted a power calculation to examine how many studies with how many participants would need to be included to assure sufficient statistical power to identify relevant effects. This power calculation was conducted according to the procedures described by Borenstein and colleagues [41]. We hoped to find a sufficient number of studies to be able to identify a small-to-moderate effect size of 0.35. These calculations indicated that we would need to include at least five studies with a mean sample size of 100 (50 participants per condition) to be able to detect an effect size of Cohen *d*=0.35 (conservatively assuming a medium level of between-study variance, T^2^, a statistical power of .80, and a significance level, alpha, of .05). Alternatively, we would need three studies with 150 participants each to detect an effect size of Cohen *d*=0.35 or nine studies with 50 participants.

### Analyses

Analyses were conducted using the statistical software Comprehensive Meta-Analysis (version 2.2.057). The effect size of subjective level of stress was calculated as the primary outcome. Furthermore, we assessed the levels of depression and anxiety when available. If more than one measure per outcome was used and if no primary outcome was indicated, then the mean of the effect size was calculated to ensure that each study yielded only one effect size. A random-effects model was chosen because of the expectations of considerable heterogeneity between studies. We further conducted a series of subgroup analyses according to the mixed-effects model. In this model, studies within subgroups are pooled with the random-effects model, whereas tests for significant differences between subgroups are conducted with the fixed-effects model. Because of the small number of studies providing follow-up data, the subgroup analyses were performed only for posttreatment and the results from the follow-ups for the primary outcome stress were clustered into two categories (1-3 months and 4-6 months).

In three studies, two treatments were compared with a single control group [36,38,42]. In these cases, we treated each comparison as a separate study, and we avoided the double counting of controls by dividing the control group in half.

The effect size in the form of Cohen *d* was used to represent the standard mean difference between the means of the intervention group and the control group at posttest. According to Cohen [43], *d*=0.2 can be considered a small effect, *d*=0.5 is a medium effect, and *d*=0.8 is a large effect. Because of the difficulty of interpreting Cohen *d* from a clinical perspective, we also transformed these values into numbers needed to treat (NNT) according to the formula of Kraemer and Kupfer [44]. The NNT indicates the number of participants who need to be treated to generate one additional clinically significant change [45].

Furthermore, we conducted the following subgroup analyses: the theoretical basis of the intervention (CBT, identified by including cognitive restructuring/challenging dysfunctional thoughts; TWC, identified by inclusion of more recent CBT-based techniques such as mindfulness, meditation, or acceptance of emotions; and alternative interventions [ALT]), guidance (guided with regular written feedback; unguided with no support or only technical support), length of the intervention (short: 1-4 weeks; medium: 5-8 weeks; long: ≥9 weeks), and the risk of bias (low risk=4; some risk<4).

Moreover, a test of homogeneity of the observed effect sizes was calculated using the *I*^2^ statistic as an indicator of heterogeneity in percentages. Thereby, a value of 0% indicates no heterogeneity, 25% is considered low, 50% is considered moderate, and 75% is considered a high level of heterogeneity [46]. We calculated 95% confidence intervals around *I*^2^ [47] using the noncentral approach based on chi-square within the heterogi module for Stata [48]. Although we calculated the Q-statistics, we only report whether the result was significant.

Publication bias was investigated by conducting a visual inspection of the funnel plot for the primary outcome measure. An asymmetric as opposed to a symmetric inverted funnel shape indicates potential publication bias that could compromise the conclusions drawn from the meta-analysis. Egger’s test [49] was used to test for the significance of the likely presence of publication bias. Additionally, we performed Duval and Tweedie’s trim-and-fill analysis [50] to verify an unbiased estimate of the pooled effect size. This analysis calculates an estimation of the number of missing studies and the potential effect of these studies on the outcome.

## Results

### Study Selection

The systematic literature research resulted in 2137 abstracts. An additional nine potentially relevant articles were identified through other searches. After removing the duplicates, we screened the titles and abstracts of 1781 articles and excluded 1687 articles because of their apparent irrelevance. With regard to eligibility, 94 full-text articles were retrieved and assessed by two independent raters (EH and DL); Cohen kappa for agreement between the independent raters was very good (Cohen κ=.83). Any discrepancies were resolved by discussion. We included 27 studies according to the inclusion and exclusion criteria. However, the results of one study [51] were accounted for in one of the other included articles [24] and it was not possible to calculate effect sizes for three studies due to insufficient data [52-54]. In three studies [42,55,56], a small proportion of the participants were younger than 18 years, which was the prespecified criterion of being classified as adults. Because the studies fit all the other inclusion criteria and our sensitivity analyses indicated no difference in the overall results, we decided to include those studies.

**Figure 1:**
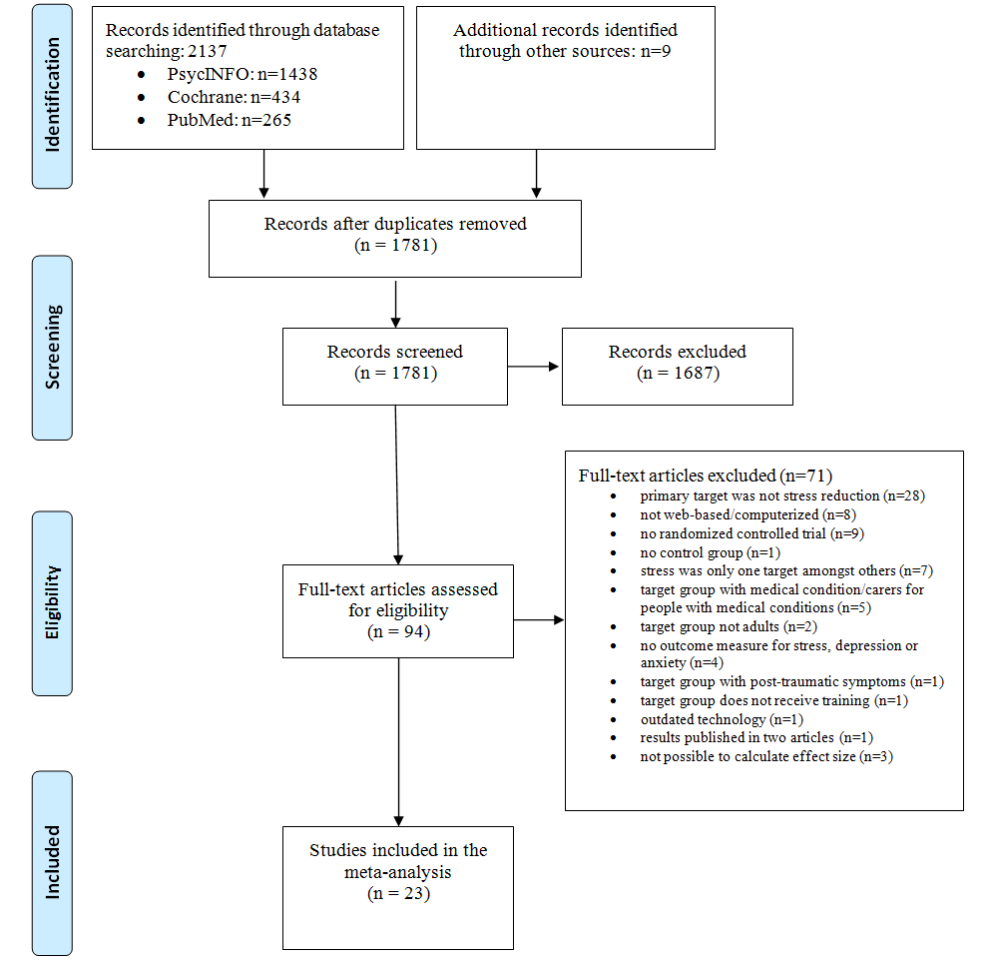
PRISMA flowchart.

Thus, we included 23 studies in the analysis. These 23 studies included 26 comparisons from baseline to posttest. Follow-up data were available for four studies (six comparisons) at 1 to 3 months and for six studies at 4 to 6 months. The PRISMA flowchart of the study selection is presented in Figure 1.

### Study Characteristics

Selected characteristics of the 23 included studies [21-39,42,55-57] are presented in Table 1 (trial characteristics) and Table 2 (intervention characteristics). A more detailed description of the interventions is available in the Multimedia Appendix 2.

The total number of participants was 4226 (intervention groups: n=2312, control groups: n=1914). The included interventions varied according to the intervention content (see Table 2 for details). Most studies evaluated interventions based on TWC (13 comparisons), followed by ALT interventions (7 comparisons), and CBT interventions (6 comparisons). Seventeen studies used a waitlist control comparison, three studies a no-treatment control group, and three studies used an attention control group. The included studies were predominantly conducted in the United States (11 studies), followed by Germany (3 studies), Sweden (2 studies), Austria/Switzerland (2 studies), Japan (2 studies), Norway (1 study), the United Kingdom (1 study), and the Netherlands (1 study).

**Table 1:** Selected trial characteristics of the included studies (N=23).

Study	Origin^a^	N^b^	Age (years), mean/mean range	Women (%)	Conditions^c^	Outcomes^d^	Risk of bias^e^	Follow-up^f^
Aikens et al (2014) [27]	US	89	N/A	N/A	TWC vs WC	Stress (PSS-14)	+? ± ??	6 m ext
Allexandre et al (2016) [28]	US	55	40.0	83.2	TWC vs WC	Stress (PSS-10)	+? ± +?	4 m; 1 y ext
Billings et al (2008) [21]	US	236	30-39	70.6	CBT vs WC	Stress (SODS); depression (CES-D); anxiety (BAI)	+? ± – ?	N/A
Cavanagh et al (2013) [29]	GB	104	24.7	88.5	TWC vs WC	Stress (PSS-10); depression (PHQ-4); anxiety (PHQ-4)	+? ± +?	N/A
Drozd et al (2013) [30]	NO	259	32.6	76.0	TWC vs WC	Stress (DASS-S)	+ + ± +?	2 m; 6 m
Ebert et al (2016) [31]	DE	263	42.0	71.5	TWC vs WC	Stress (PSS-10); depression (CES-D); anxiety (HADS-A)	+? ± + +	6 m
Ebert et al (2016) [32]	DE	263	42.9	85.9	TWC vs WC	Stress (PSS-10); depression (CES-D) anxiety (HADS-A)	+? ± + +	6 m
Frazier et al (2015) [56]	US	194	18-21	75.0	ALT vs AC	Stress (PSS-10, DASS-S); depression (DASS-D) anxiety (DASS-A)	+? ± +?	5 w
Glück & Maercker (2011) [33]	AT, CH	47	35.2	73.5	TWC vs WC	Stress (PSQ)	+ – ± +?	3 m ext
Hänggi (2006) [22]	CH	125	25-45	85.0	CBT vs WC	Stress (LOS)	+ + ± – ?	1 m ext; 6 m ext
Heber et al (2016) [34]	DE	264	43.3	73.1	TWC vs WC	Stress (PSS-10); depression (CES-D); anxiety (HADS-A)	+? ± + +	6 m; 1 y ext
Hinman et al (1997) [39]	US	50	37.7	100.0	ALT vs NT	Stress (PNQ (combined)	+? ± ? ?	N/A
Hintz et al (2015) [42]	US	204	18-21	70.0	ALT vs AC; ALT vs AC (II)	Stress (PSS-10, DASS-S); depression (DASS-D); anxiety (DASS-A)	+? ± – ?	5 w
Ly et al (2014) [35]	SE	73	41.5	42.5	TWC vs WC	Stress (PSS-14)	+ + ± + ?	N/A
Morledge/ Allexandre et al (2013) [36]	US	279	40-59	88.9	TWC vs TWC+OMB vs WC	Stress (PSS-10)	+ + ± – ?	1 m
Nguyen-Feng et al (2015) [55]	US	500	18-21	62.0	ALT vs WC	Stress (PSS-10, DASS-S); depression (DASS-D); anxiety (DASS-A)	+ ? ± + ?	N/A
Rose et al (2013) [23]	US	59	27.3	50.0	CBT vs AC	Stress (PSS-10)	+ ? ± – ?	N/A
Ruwaard et al (2007) [24]	NL	239	44.0	60.0	CBT vs WC	Stress (DASS-S); depression (DASS-D); anxiety (DASS-A)	+ ? ± + ?	3 y ext
Umanodan et al (2014) [25]	JP	263	38.9	7.2	CBT vs WC	Stress (BJSQ)	+ ? ± + ?	19 w
Wiegand et al (2010) [38]	US	562	35.8	100.0	ALT vs TWC vs NT	Stress (PSS-10); anxiety (STAI)	+ ? ± – ?	N/A
Wolever et al (2012) [37]	US	105	42.9	76.6	TWC vs NT	Stress (PSS-10); depression (CES-D)	+ + ± + ?	N/A
Yamagishi et al (2008) [57]	JP	36	33.0	N/A	ALT vs WC	Stress (JSBQ combined); depression (BJSQ-D); anxiety (BJSQ-A)	+ ? ± – ?	1 m ext
Zetterqvist et al (2003) [26]	SE	63	39.2	61.9	CBT vs WC	Stress (PSS-14); depression (HADS-D); anxiety (HADS-A)	+ ? ± – ?	N/A

^a^AT: Austria; CH: Switzerland; DE: Germany; GB: United Kingdom; JP: Japan; NL: the Netherlands; NO: Norway; SE: Sweden; US: United States of America.

^b^Indicates only the number of participants included in this analysis.

^c^AC: attention control group; ALT: alternative; CBT: cognitive behavioral therapy; NT: no treatment; OMB: online message board; TWC: third-wave cognitive behavioral therapy; WC: waitlist control.

^d^BAI: Beck Anxiety Inventory; BJSQ-A: Brief Job Stress Questionnaire-Anxiety Subscale; BJSQ-D: Brief Job Stress Questionnaire-Depression Subscale; CES-D: Center for Epidemiologic Studies Depression Scale; DASS-A: Depression Anxiety Stress Scales-Anxiety Subscale; DASS-D: Depression Anxiety Stress Scales-Depression Subscale; DASS-S: Depression Anxiety Stress Scales-Stress Subscale; HADS-A: Hospital Anxiety and Depression Scales-Anxiety Subscale; HADS-D: Hospital Anxiety and Depression Scales-Depression Subscale; JSBQ: Job Stress Brief Questionnaire; LOS: Level of Stress (self-created instrument); PHQ-4: Patient Health Questionnaire for Depression and Anxiety; PNQ: Personal Strain Questionnaire; PSQ: Perceived Stress Questionnaire; PSS-10: Cohen’s Perceived Stress Questionnaire (10-item version); PSS-14: Cohen’s Perceived Stress Questionnaire (14-item version); SODS: Symptoms of Distress Scale; STAI: State Trait Anxiety Inventory.

^e^Risk of bias was judged according to the following criteria: (1) adequate sequence generation, (2) allocation concealment, (3) blinding (± indicates that only self-reported data were used), (4) adequate consideration of incomplete data, and (5) prevention of selective outcome. +: no bias; –: bias; ?: information was insufficient to make judgments.

^f^Ext: extended follow-up; m: months; w: weeks; y: years.

**Table 2:** Selected intervention characteristics of included studies.

Study	Label and content	Type^a^	Guidance^b^	Delivery	Length (weeks)
Aikens et al (2014) [27]	Mindfulness goes to work: Mindfulness program combined live, instructor-led, weekly hour-long virtual meetings (webinar) with online applied training.	TWC	G	Web	7
Allexandre et al (2016) [28]	Stress Free Now / Online Mindfulness Program for Stress Management: Interactive, educational program based on mindfulness meditation. Includes exercises, email reminders, and downloads.	TWC	UG (R)	Web	8
Billings et al (2008) [21]	Stress and Mood Management Intervention: Array of CBT techniques. Entire program is audio-narrated with the use of videos and graphics.	CBT	UG	Web	12
Cavanagh et al (2013) [29]	Learning Mindfulness Online: Daily, 10-min guided mindfulness meditation audio tracks delivered via a virtual learning facility (Moodle). Four reminder emails.	TWC	UG (R)	Web	2
Drozd et al (2013) [30]	Less Stress intervention: Eclectic approach that included mindfulness and metacognitive exercises with 13 short modules. Hyperlinks sent via email to the participants.	TWC	UG	Web	4
Ebert et al (2016) [31]	GET.ON Stress Self-Guided: Theory-based intervention focusing on problem solving and emotion regulation. Tailored to employees; optional text message coaching.	TWC	UG	Web	7
Ebert et al (2016) [32]	GET.ON Stress Adherence-Focused Guided: Theory-based intervention focusing on problem solving and emotion regulation. Tailored to employees; optional text message coaching; written feedback on request; reminders.	TWC	AFG	Web	7
Frazier et al (2015) [56]	Present Control Intervention: Theory-based intervention focused on perceived control; 4 modules over 2-week period. Included stress logs and reminder emails.	ALT	UG (R)	Web	2
Glück & Maercker (2011) [33]	Brief Web-based mindfulness training: 2 modules. 20-minute units per day, audio files, a flash animated exercise, and written text.	TWC	UG (R)	Web	2
Hänggi (2006) [22]	Online parental training on coping with family stress: 4 modules (eg, cognitive restructuring, time management, muscle and breathing relaxation, problem solving).	CBT	UG	Web	4
Heber et al (2016) [34]	GET.ON Stress Guided: Theory-based intervention focusing on problem solving and emotion regulation. Tailored to employees; optional text message coaching; written feedback; reminders.	TWC	G	Web	7
Hinman et al (1997) [39]	Exercise Break: 2 × 15 minutes per day. Stretching, circulatory and relaxation exercises. Accessed via local computer network at the workplace.	ALT	UG	PC	8
Hintz et al (2015) [42]	Present Control Intervention: Theory-based intervention focused on perceived control; 4 modules over 2-week period. Included stress logs and reminder emails. Group I: with personalized feedback, Group II: unguided.	ALT	UG (R) & G	Web	2
Ly et al (2014) [35]	Acceptance- and commitment-based mobile phone app: step-by-step behavior program including 6 basic principles to handle stress. 15 min daily. Short writing reflection. One-way therapist-client support through text messages every other day.	TWC	G	Web (smartphone)	6
Morledge/Allexandre et al (2013) [36]	Online Mindfulness Program for Stress Management: Eight mindfulness modules consisting of introduction, meditations, articles, and tips and exercises. Group II: program plus online message board.	TWC	UG (R)	Web	8
Nguyen-Feng et al (2015) [55]	Present Control Intervention: Theory-based intervention focused on perceived control; 3 modules: psycho-educational video of a professor, animated video (Prezi), and a written exercise. Includes stress logs and reminders.	ALT	UG (R)	Web	5
Rose et al (2013) [23]	Self-guided, multimedia stress management and resilience training program, SMART-OP: consists of at least one exercise in each of 3 domains: feelings, thoughts, and actions. Includes game-like activities.	CBT	UG (R)	PC	6
Ruwaard et al (2007) [24]	Emailed Standardized CBT of Work-Related Stress: 7 modules (eg, relaxation, challenging dysfunctional thoughts, time management). 10 feedbacks/5 hours of therapist time.	CBT	G	Web	7
Umanodan et al (2014) [25]	SMT program in employees: Self-paced program. (1) behavioral techniques, (2) communication techniques, and (3) cognitive techniques; skill acquisition and practice phase; weekly emails.	CBT	UG (R)	PC	7
Wiegand et al (2010) [38]	Comprehensive program for reducing stress: Group I: Daily use of olfactory care products plus an Internet-based program focusing on stress reduction, prevention and behavioral modification. Periodic feedback reports are provided. Group II: Internet-based program only.	ALT, TWC	UG	Web	12
Wolever et al (2012) [37]	Mindfulness at Work Intervention: Virtual classroom with real-time bidirectional communication with an experienced mindfulness trainer (12 modules, 14 hours). Includes brief exercises designed to be used at work.	TWC	G	Web	12
Yamagishi et al (2008) [57]	Web-based career identity training for stress management: 4 modules. (1) definition of career identity, (2): cognition of own career identity, (3): characteristics of nurses’ career identity, (4): career goal management and planning.	ALT	UG	Web	3
Zetterqvist et al (2003) [26]	Internet-based self-help stress-management program: Each module consists of 3 sections: relaxation, additional exercises (eg, problem solving), and information (eg stress at work). Exercises were sent in and participants received feedback as a prompt to continue; includes reminders.	CBT	G	Web	6

^a^ALT: alternative; CBT: cognitive behavioral therapy; TWC: third-wave cognitive behavioral therapy.

^b^AFG: Adherence-focused guidance; G: guided; UG: unguided; UG (R): unguided with reminders via mail or telephone.

The interventions of the included studies were mainly Web-based interventions (n=20). For Web-based interventions, an active Internet connection is necessary (eg, to access a website, use a mobile phone app, or visit a virtual classroom). A total of three computer-based interventions [23,25,39] were found in which interventions were installed, for example, on a desktop computer in a separate room at work.

The Perceived Stress Scale (PSS-10, PSS-14) was predominantly used to assess the level of stress (15 of 23 studies). Follow-up assessments have been reported for nine studies (12 comparisons); whereas four studies (6 comparisons) reported data for up to 3 months and six studies (6 comparisons) for 4 to 6 months (see Table 1). Extended follow-ups (no comparison with the respective control groups) were conducted at 1 month (2 studies), 3 months (1 study), 6 months (2 studies), one year (2 studies), and 3 years (1 study). For nine studies, only posttest assessments were available. The participants received guidance in seven studies [24,26,27,34,35,37,42], one study [32] assessed a less intensive guidance format (adherence-focused guidance: combination of reminders and written feedback only on request of the participants), and 16 studies (18 comparisons) investigated unguided interventions. Nine unguided studies reported the use of automated or telephone and mail reminders for completion of the intervention [23,25,28,29,33,36,42,55,56] (Table 2).

### Risk of Bias Within Studies

A risk of bias was present in all studies. Only six studies fulfilled four of the five criteria used. Nine studies met three criteria, and eight studies fulfilled two criteria. In most cases, the concealment of allocation was insufficiently described, and only 13 of 23 studies reported adequate handling of missing data. In particular, the risk for selective outcome reporting was unclear because the study registration prior to the trials could only be retrieved from three studies [31,32,34]. Although another four studies registered their trial [28,30,33,36], this step occurred retrospectively. The mean interrater reliability between independent raters was κ=.84 and ranged from .60 (selective outcome reporting) to 1.0 (blinding). Disagreements were handled by discussion.

### Publication Bias

Neither the inspection of the funnel plot nor the Egger’s test [49] indicated a possible publication bias. Moreover, the Duval and Tweedie trim-and-fill analysis [50] indicated no missing studies.

### Effects on Levels of Stress, Depression, and Anxiety

**Figure 2:**
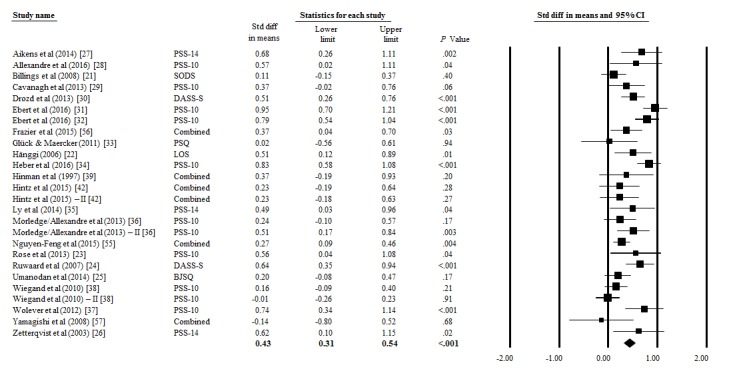
All effect sizes included in the meta-analysis from the studies comparing computer- and Web-based stress-management interventions to a control group. BJSQ: Brief Job Stress Questionnaire; DASS-S: Depression Anxiety Stress Scales-Stress Subscale; LOS: Level of Stress (self-created instrument); PSQ: Perceived Stress Questionnaire; PSS-10: Cohen’s Perceived Stress Questionnaire (10 item version); PSS-14: Cohen’s Perceived Stress Questionnaire (14 item version); SODS: Symptoms of Distress Scale.

Table 3 presents the effect sizes, confidence intervals, level of significance and heterogeneity for the overall effects on stress, depression, and anxiety as well as for the subgroups. The overall analysis of effect sizes yielded a significant effect size of Cohen *d*=0.43 for the primary outcome stress across all studies (95% CI 0.31-0.54; n=26). Heterogeneity was moderate (*I*^2^=68.01, 95% CI 52.08-78.72). Significant small effect sizes were observed for the secondary outcomes depression (Cohen *d*=0.34, 95% CI 0.21-0.48; n=13) and anxiety (Cohen *d*=0.32, 95% CI 0.17-0.47; n=14). Figure 2 displays a forest plot of the effect sizes and the confidence intervals of the included studies.

**Table 3:** Effects of computer- and Web-based stress-management interventions for healthy adults compared to control groups.

Study			Comparisons, n	Cohen *d* (95% CI)	*P*	*Z*	*I*^2^ (95% CI)	*P* ^a^	NNT	*P* ^b^
**Overall effect**										
	**Primary outcome**									
		Stress at posttest	26	0.43 (0.31, 0.54)	<.001	7.12	68.01 (52.08, 78.72)	<.001	4.20	
		1-3 m follow-up	6	0.33 (0.19, 0.46)	<.001	4.60	0.00 (0.00, 74.62)	.55	5.43	
		4-6 m follow-up	6	0.56 (0.25, 0.87)	<.001	3.55	85.93 (71.44, 93.07)	<.001	3.25	
	**Further outcomes (posttest)**									
		Depressive symptoms	13	0.34 (0.21, 0.48)	<.001	4.93	58.25 (22.81, 77.41)	.004	5.26	
		Anxiety symptoms	14	0.32 (0.17, 0.47)	<.001	4.16	71.13 (50.34, 83.22)	<.001	5.56	
**Risk of bias score^c^**										
	Low risk		6	0.74 (0.59, 0.89)	<.001	9.82	35.44 (0.00, 74.20)	.17	2.50	<.001
	Some risk		20	0.30 (0.21, 0.40)	<.001	6.26	31.43 (0.00, 60.17)	.09	5.95	
**Theoretical basis^c^**										
	CBT		6	0.40 (0.19, 0.61)	<.001	3.75	52.68 (0.00, 81.12)	.06	4.50	.03
	TWC		13	0.53 (0.35, 0.71)	<.001	5.67	74.50 (55.98, 85.23)	<.001	3.42	
	ALT		7	0.24 (0.12, 0.36)	<.001	4.03	0.00 (0.00,70.81)	.85	7.46	
**Guidance^c,d^**										
	Yes		7	0.64 (0.50, 0.79)	<.001	8.53	11.81 (0.00, 74.24)	.34	2.86	.002
	No		18	0.33 (0.20,0.46)	<.001	5.02	62.72 (38.03, 77.57)	<.001	5.43	
**Length^c^**										
	Short		9	0.33 (0.22, 0.44)	<.001	5.94	0.00 (0.00, 64.80)	.56	5.43	.006
	Medium		13	0.59 (0.45, 0.74)	<.001	7.89	54.92 (15.83, 75.86)	.008	3.09	
	Long		4	0.21 (-0.05, 0.47)	.11	1.61	71.00 (17.25, 89.85)	.02	8.47	

^a^The *P* values indicate whether the Q-statistic is significant (the *I*^2^ statistics do not include a test of significance).

^b^This *P* value indicates whether the differences between subgroups were significant.

^c^Subgroup calculations for the primary outcome stress.

^d^One study [32] was excluded because it could not be classified.

### Subgroup Analyses

#### Risk of Bias

Accounting for the risk of bias level was associated with a considerable reduction of heterogeneity: studies with a lower risk of bias showed low heterogeneity (*I*^2^=35.44, 95% CI 0.00-74.20). Subgroup analyses revealed that studies at low risk produced significantly larger effect sizes (Cohen *d*=0.74, 95% CI 0.59-0.89; n=6) than did studies with some risk (Cohen *d*=0.30, 95% CI 0.21-0.40; n=20; *P*<.001).

#### Theoretical Basis

The subgroup analysis of the theoretical basis of the interventions was significant (*P*=.03) and showed that TWC interventions produced a highly significant medium effect size of Cohen *d*=0.53 (95% CI 0.35-0.71; n=13). CBT interventions led to reductions in stress levels with a highly significant effect size of Cohen *d*=0.40 (95% CI 0.19-0.61; n=6). In contrast, ALT interventions produced a small effect size (Cohen *d*=0.24; 95% CI 0.12-0.36; n=7).

#### Guidance

With regard to the subgroup guidance, the results show that guided interventions (Cohen *d*=0.64, 95% CI 0.50-0.79; n=7) were significantly more effective than unguided interventions (Cohen *d*=0.33, 95% CI 0.20-0.46; n=18; *P*=.002).

#### Length

A significant difference was also found for the length of the interventions (*P*=.006). We found significant small-to-medium effect sizes for short (Cohen *d*=0.33, 95% CI 0.22-0.44; n=9) and medium interventions (Cohen *d*=0.59, 95% CI 0.45-0.74; n=13), whereas long interventions led to a nonsignificant effect size of Cohen *d*=0.21 (95% CI –0.05 to 0.47; n=4; *P*=.11).

#### Follow-up

Results of the 1 to 3 month follow-ups for the primary outcome stress showed a small effect size of Cohen *d*=0.33 (95% CI 0.19-0.46; n=6) and, for the 4 to 6 month follow-ups, a medium effect size of Cohen *d*=0.56 (95% CI 0.25-0.87; n=6).

## Discussion

The aim of this paper was to conduct a meta-analysis of Web- and computer-based interventions for stress management in adults. Our analyses yielded four main findings. First, Web- and computer-based stress-management interventions can be effective in reducing stress, depression, and anxiety and maintain those effects for up to 6 months. Second, interventions using TWC and CBT interventions as a theoretical basis proved moderately effective in reducing stress. Third, short and medium interventions (up to 8 weeks) are more effective than long interventions (9 weeks and longer). Fourth, guided interventions yielded a greater effect size for reduction of stress than unguided interventions.

For the primary outcome stress, an effect size of Cohen *d*=0.43 was found across the 26 comparisons. Small effect sizes were found for depression (Cohen *d*=0.34) and anxiety (Cohen *d*=0.32). A recent synthesis of five meta-analyses of traditional stress-management interventions found a between-group overall mean effect size of Cohen *d*=0.45 (95% CI 0.41-0.48) [58], which is comparable to the effect of stress, but somewhat higher for the effects of depression and anxiety found in this meta-analysis. To date, no trials have been conducted that aim to assess the equivalence of face-to-face and Web-based stress-management interventions in a methodologically robust design. One trial comparing an online versus face-to-face version of stress management in a single trial indicates that there is no difference in reductions of stress or depression levels [37]. Nevertheless, higher effect sizes were also found in Richardson and Rothstein’s work [9] concerning individual outcome measures in traditional stress-management interventions, particularly for stress (Cohen *d*=0.73) and anxiety (Cohen *d*=0.68). Therefore, it may be possible that traditional interventions yield slightly higher effect sizes. One possible explanation for this is that face-to-face interventions are superior in reducing these outcomes. An alternative explanation is that Web-based interventions may reach affected individuals at an earlier stage, with lower baseline levels and thus less room for improvement. In fact, most studies included in the present work did not use a cut-off on a relevant stress scale, and most of those that did (eg, [23,37,38]) used a relatively low cut-off threshold. It may be the case that participants who are more severely impaired might generally prefer face-to-face over Web-based interventions and that this effect is reflected in greater improvements stemming from their higher baseline stress levels. This is in line with the fact that the highest effect sizes in this meta-analysis were produced from trials targeting highly stressed individuals [31,32,34]. Evidence on a Web-based depression intervention also showed that a higher severity of baseline scores significantly predicted better treatment outcomes [59]. Although the effect sizes found here are somewhat smaller than those found in traditional face-to-face interventions, Web- and computer-based interventions can have greater reach. At the population level, even small-to-moderate effects can have a substantial influence. More research is needed to clarify the effects of face-to-face and Web-based stress-management interventions in direct comparisons.

Based on the assumption that the treatment effect varied as a function of other factors, we conducted a number of subgroup analyses. First, we investigated the effect of guidance. The results showed that guided interventions (Cohen *d*=0.64) are significantly more effective than unguided interventions (Cohen *d*=0.33), with effect sizes for guided interventions comparable to traditional face-to-face interventions. The finding of guided interventions being superior to unguided interventions is consistent with results on Web-based interventions for other conditions, such as depression and anxiety [60,61]. Providing support to clients in terms of weekly feedback may enhance adherence to the intervention and thus improve treatment efficacy [62]. Such an assumption is in line with a study on pooled data from three RCTs showing that guidance was associated with greater adherence rates in Internet-based stress-management compared to unguided interventions [63]. The finding that guided interventions are more effective than unguided interventions is consistent with a systematic review that found guided Web-based mental health interventions to be significantly superior to unguided interventions [61]. The results in this analysis indicate an advantage for guided interventions, although it is unclear how much guidance and in what manner it produces the largest effect sizes. One trial in this meta-analysis used an alternative, more economic format of guidance (adherence-focused guidance; ie, feedback only on request plus weekly reminders) and produced large effect sizes at posttest and 6-month follow-up [32], and the adherence rates were comparable to a more intensive guidance format using the same intervention [63]. More research on the relative level and type of human involvement needed in these interventions would be useful [64].

Consistent with existing evidence on face-to-face interventions [8,9], the CBT interventions included in this study were efficacious. The effect sizes of CBT (Cohen *d*=0.40) and TWC interventions (Cohen *d*=0.53) were smaller than the average effect size found in two meta-analyses for traditional CBT interventions (Cohen *d*=0.68 [8]; Cohen *d*=1.16 [9]), although, on average, the effect sizes fall within the confidence interval of the latter work [9]. Direct comparisons examining the relative efficacy of the two training formats would be needed to draw firm conclusions. Due to their proliferation in the last few years, TWC interventions were introduced as a new category in this meta-analysis as they extend the traditional CBT techniques with newer “third-wave” components, such as acceptance of emotions or mindfulness. These interventions have been found to be effective in alleviating symptoms that are associated with a wide range of physical, psychosomatic, and psychiatric disorders [65,66], including stress [67]. Comparable to our findings, early evidence on face-to-face interventions suggests that TWC and traditional CBT approaches are equally effective and acceptable in the treatment of acute depression [68]; nevertheless, more high-quality studies are needed to support this assumption. As opposed to (third-wave) CBT interventions, alternative approaches (eg, career identity training, combination with olfactory care products) only produced a small effect size.

A significant between-group effect was also found for the length of the intervention. In contrast to short-to-medium interventions, long interventions (9 weeks and longer) were not found to be effective. One possible explanation is that it may be more difficult for participants to remain engaged in longer interventions compared with shorter interventions. These results correspond to findings from the area of depression, in which shorter interventions have been found to be more effective than longer interventions [61]. Research on the relevance of treatment intensity suggests that the number of therapy sessions is not related to the outcome and keeping the number of sessions equal, but providing the sessions over a shorter period of time, may be associated with better treatment outcomes [69]. Nevertheless, because there were only four comparisons available for long interventions, this conclusion should be interpreted with care. Future research should examine the optimal intensity and length of interventions.

### Limitations

This meta-analysis has a number of limitations. First, because the risk of bias in the included studies was high, these results must be interpreted with caution. Second, the overall number of studies for the follow-up points and for some subgroups is small, limiting the strength of conclusions that can be drawn from these results. Third, we found a moderate degree of heterogeneity for the primary outcome that was reduced when analyzing the level of risk of bias and guidance subgroups; nevertheless, the number of comparisons in some subgroups was small and did occasionally overlap concerning individual studies. Fourth, we are mindful of the possibility that despite our thorough literature search, we might have missed a relevant study. Finally, because of the lack and inconsistency of information provided by the included studies, we were unable to analyze the effect of potentially relevant moderators of the treatment effect, such as the effect of adherence to the intervention on the overall outcome.

### Future Recommendations

In future studies, preregistration of trials, an adequate calculation of the sample size, a more detailed description of allocation concealment, and appropriate methods to account for missing values are strongly recommended. We observed that a growing number of studies adhered to the intention-to-treat principle (ie, by employing mixed-effects models); nevertheless, especially for those studies, we recommend to ensure that the reported descriptive statistics are based on adequate methods to handle missing values and do not solely rely on complete cases. This will ensure adherence to the intention-to-treat principle throughout all statistics.

Although in most studies the therapeutic approach that was used in the intervention (eg, CBT) is often well described, it would be desirable for future studies to also specify the theoretical model that was used to develop the intervention (eg, conservation of resources theory, transactional model of stress).

Overall, more methodologically rigorous studies with a low risk of bias are needed to assess the effect of, for example, particular characteristics of interventions, such as treatment latitude, different levels of guidance, and different types of interventions in a direct comparison. Moreover, information on the number of participants who adhered to the intervention and details on co-interventions alongside training would be insightful. Future research should also test Web- and computer-based stress management interventions against the highest standard in this field (ie, classical face-to-face stress management interventions) and should more frequently include longer follow-up periods (eg, up to 6 months). Data on the cost-effectiveness of such interventions would also be highly relevant.

### Conclusions

Despite the limitations discussed, it appears safe to conclude that Web- and computer-based interventions can be effective. In particular, interventions that include guidance from an online coach, are of medium length, or that are based on (third-wave) CBT lead to moderate improvements in stress levels. Initial evidence also suggests that the effects can be maintained up to 6 months. Whereas research and practice on traditional face-to-face interventions have been prolific [8,9], research on the efficacy and dissemination of Web-based stress-management interventions is still at the beginning despite the high potential and reach of such interventions. This work draws attention to the need for further studies on the efficacy, cost-effectiveness, and mechanisms of change of such interventions. In summary, the integration of evidence-based Web-based stress management interventions into health care systems has the potential to make a valuable contribution to reducing stress-related mental health problems on a large scale.

## Appendix

Multimedia Appendix 1 Search strategy.

Multimedia Appendix 2 Selected intervention characteristics of included studies.

## Abbreviations

CBT: cognitive behavioral therapy
NNT: number needed to treat
RCT: randomized controlled trial
TWC: third-wave cognitive behavioral therapy
