# Supplementary material for: The Benefit of Web- and Computer-Based Interventions for Stress: A Systematic Review and Meta-Analysis
Source: J Med Internet Res. 2017 Feb 17;19(2):e32. doi: 10.2196/jmir.5774 (PMC5336602; doi:10.2196/jmir.5774)
Supplement: Multimedia Appendix 1 [file jmir_v19i2e32_app1.pdf]

Multimedia Appendix 1. Search strategy.

Search terms	Stress	*AND*	Intervention	*AND*	Design	*AND*	Online Context
Limitation in database	All fields		Abstract		Abstract		Abstract
Keywords	Stress		Intervention (OR)<br>Program (OR)<br>Programme (OR)<br>Training		RCT (OR)<br>Randomised (OR)<br>Randomized (OR)<br>Randomly (OR)<br>Pre (OR)<br>Post (OR)<br>Eval* (OR)<br>Effect* (OR)<br>Control* (OR)<br>Compar* (OR)<br>Outcome*		Internet (OR)<br>Web (OR)<br>Online (OR)<br>Computer-aided (OR)<br>Computer-assisted (OR)<br>Computer-guided (OR)<br>Computer-driven (OR)<br>Computerized (OR)<br>Computerised

Source:

Heber E, Ebert DD, Lehr D, Cuijpers P, Berking M, Nobis S, Riper H. The Benefit of Web- and Computer-Based Interventions for Stress: A Systematic Review and Meta-Analysis. J Med Internet Res 2017;19(2):e32
