# Supplementary material for: The Benefit of Web- and Computer-Based Interventions for Stress: A Systematic Review and Meta-Analysis
Source: J Med Internet Res. 2017 Feb 17;19(2):e32. doi: 10.2196/jmir.5774 (PMC5336602; doi:10.2196/jmir.5774)
Supplement: Multimedia Appendix 2 [file jmir_v19i2e32_app2.pdf]

Multimedia Appendix 2. Selected intervention characteristics of included studies.

Study (Label)	Content	Type[a]	Guidance[b]	Delivery	Length (weeks)
Aikens et al (2014) [27]	Mindfulness goes to work: Program combined live, instructor-led, weekly hour-long virtual class meetings (webinar) with accompanying online applied training. Access to unique training dashboard and practice guide workbook. Training in mindfulness practices: (1) seated focus exercises, (2) body scan, (3) walking meditation, (4) movement exercise, (5) 3-minute breathing pause. Additional exercises included performance-oriented skills (eg mindful problem solving). Included preprogrammed email coaching, progress tracking survey, and optional text messaging system (practice reminders and encouragement).	TWC	G	Web	7
Allexandre et al (2016) [28]	Stress Free Now / Online Mindfulness Program for Stress Management: Interactive, educational program based on mindfulness meditation. Each of the 8 modules consists of lesson introduction, meditations, articles, and tips. Exercises include body scan, mindfulness meditation, guided imagery, letting go and forgiveness, confidence and self-esteem meditation, loving kindness meditation, mystery and miracles of life, and a still mind. Twice a week email reminders to access the website and practice meditation. Introductory talks and meditation exercises were also provided on CDs in mp3 format.	TWC	UG (R)	Web	8
Billings et al (2008) [21]	Stress and Mood Management Intervention: Array of CBT techniques, such as goal setting, problem solving, identifying and testing negative thoughts, relaxation, and time management. Stress management as a gateway for other content (substance misuse, anxious mood, and dysphoria). Entire program is audio-narrated with the use of videos and graphics.	CBT	UG	Web	12
Cavanagh et al 2013 [29]	Learning Mindfulness Online: Daily, 10-min guided mindfulness meditation audio tracks delivered via a virtual learning facility (Moodle). Audio guidance included an invitation to bring a nonjudgmental awareness to bodily sensations, the breath, thoughts, and feelings. Program had 5 sections: (1) What is Mindfulness? (2) Daily Mindfulness Practice, (3) Daily Practice FAQ, (4) My Daily Journal, (5) Study Information, Help, and Assistance. Included 4 standardized reminder emails in the 2-week training period.	TWC	UG (R)	Web	2
Drozd et al (2013) [30]	Less Stress intervention: 13 modules. Eclectic approach that included mindfulness and metacognitive exercises. Hyperlinks sent via email to the participants, with 10 minutes to complete. Each session has 2 components: (1) stress-related topic; (2) techniques, exercises, and homework.	TWC	UG	Web	4

Study (Label)	Content	Type[a]	Guidance[b]	Delivery	Length (weeks)
Ebert et al (2016) [31]	GET.ON Stress Self-Guided: 7 modules plus booster session. (1) psychoeducation, (2-3) problem solving (6-step systematic procedure), (4-6) emotion regulation (muscle & breathing relaxation, acceptance of emotions, emotional self-support), (7) plan for the future; based on Lazarus' transactional stress model; included optional text message coaching (eg, short exercises) and additional stress-related topics; was tailored to employees; included interactive tasks, video and audio files, and a stress diary; strong focus on homework application and booster session 4 weeks after training completion; completely self-guided.	TWC	UG	Web	7
Ebert et al (2016) [32]	GET.ON Stress Adherence-Focused Guided: 7 modules plus booster session. (1) psychoeducation, (2-3) problem solving (6-step systematic procedure), (4-6) emotion regulation (muscle-& breathing relaxation, acceptance of emotions, emotional self-support), (7) plan for the future; based on Lazarus' transactional stress model; included optional text message coaching (eg, short exercises) and additional stress-related topics; was tailored to employees; included interactive tasks, video and audio files, and a stress diary; strong focus on homework application and booster session 4 weeks after training completion; written feedback from a psychologist only on request, weekly reminders if necessary.	TWC	AFG	Web	7
Frazier et al (2015) [56]	Present Control Intervention: 4 modules over 2-week period. Modules 1-3: expert video explaining theoretical background, narrated video presentation with former participants, and an application exercise. (1) information about common college student stressors and their effects; (2) definition of past, present, and future control and description of the positive outcomes related to present control; (3) avoiding pitfalls when focusing on present control; (4) continuous application of skills (video only). Included stress logs to increase motivation and reminder emails.	ALT	UG (R)	Web	2
Glück & Maercker (2011) [33]	Brief Web-based mindfulness training: 2 modules. 20-minute units per day, audio files, a flash animated exercise, and written text. (1) guided mindfulness exercises (ie, awareness of body sensations, attention to breath and acceptance of upcoming emotions); (2) distressing thoughts, feelings or sensations, to label these cues nonjudgmentally and imagine placing them on a cloud while watching it float away.	TWC	UG (R)	Web	2
Hänggi (2006) [22]	Online parental training on coping with family stress: 4 modules: (1) individual stress (eg, cognitive restructuring, time management, muscle and breathing relaxation); (2) communication; (3) family stress; (4) problem solving.	CBT	UG	Web	4

Study (Label)	Content	Type[a]	Guidance[b]	Delivery	Length (weeks)
Heber et al (2016) [34]	GET.ON Stress Guided: 7 modules plus booster session. (1) psychoeducation, (2-3) problem solving (6-step systematic procedure), (4-6) emotion regulation (muscle & breathing relaxation, acceptance of emotions, emotional self-support), (7) plan for the future; based on Lazarus' transactional stress model; included optional text message coaching (eg, short exercises) and additional stress-related topics; was tailored to employees; included interactive tasks, video and audio files, and a stress diary; strong focus on homework application and booster session 4 weeks after training completion; written feedback on each session from a psychologist, weekly reminders if necessary.	TWC	G	Web	7
Hinman et al (1997) [39]	Exercise Break: 2 × 15 minutes per day. Stretching, circulatory and relaxation exercises. Morning session: general warm-up, back, wrist, and finger exercises. Afternoon session: neck, leg, shoulder, relaxation, and circulation exercises. Accessed via local computer network at the workplace.	ALT	UG	PC	8
Hintz et al (2015) [42]	Present Control Intervention: 4 modules over 2-week period. Modules 1-3: Expert video explaining theoretical background, narrated video presentation with former participants, and an application exercise. 1: information about common college student stressors and their effects; 2: definition of past, present, and future control and description of the positive outcomes related to present control; 3: avoiding pitfalls when focusing on present control; 4: continuous application of skills (video only). Included stress logs to increase motivation and personalized reminder emails. Group I: with personalized feedback, Group II: unguided.	ALT	UG (R) & G	Web	2
Ly et al (2014) [35]	Acceptance- and commitment-based mobile phone app: The smartphone intervention consisted of a step-by-step behavior program with the purpose of educating the participant to use six basic principles to handle their stress. 6 weekly modules: Each module consisted of a short audio lecture (4-6 min), 2-3 texts and 2-4 exercises. Recommended use: 15 min daily. Short writing reflection after completion. One-way therapist-client support through encouraging text messages every other day.	TWC	G	Web (smartphone)	6
Morledge/ Allexandre et al (2013) [36]	Online Mindfulness Program for Stress Management: Each of the 8 modules consists of lesson introduction, meditations, articles, and tips. Exercises include body scan, mindfulness meditation, guided imagery, letting go and forgiveness, confidence and self-esteem meditation, loving kindness meditation, mystery and miracles of life, and a still mind. Group II: program plus online message board.	TWC	UG (R)	Web	8

Study (Label)	Content	Type[a]	Guidance[b]	Delivery	Length (weeks)
Nguyen-Feng et al (2015) [55]	Present Control Intervention: 3 modules each consisting of: psychoeducational video of a professor, animated video created using Prezi with examples from others, and a written exercise. (1) research on stress and its effects on college students' mental and physical health and academic performance. (2&3) describing different aspects of control and how they are related to adjustment (focus on present control). Focus on application of lesson content to their own lives. Last step: completion of 6 stress logs that involved writing about current stressors, assessment what aspects are controllable or uncontrollable, possible actions regarding the controllable aspects, and changes about the stressors as a result of the actions they had taken (after the first stress log).	ALT	UG (R)	Web	5
Rose et al (2013) [23]	Self-guided, multimedia stress management and resilience training program, SMART-OP: Each of the 6 modules consists of at least one exercise in each of 3 domains: feelings (eg, relaxation), thoughts (eg, cognitive restructuring), and actions (eg, problem solving). Contains animations, game-like activities, and interactive didactic videos. Strong focus on homework/application of skills between sessions (received printouts, flash drive).	CBT	UG (R)	PC	6
Ruwaard et al (2007) [24]	Emailed Standardized CBT of Work-Related Stress: 7 modules. (1) inducing awareness, (2) relaxation, (3) worrying/ruminating and challenging dysfunctional thoughts, (4) positive self-verbalization, (5) positive assertiveness/social skills training and behavioral experiments, (6) time management, (7) future, reintegration and relapse prevention. 10 feedbacks/5 hours of therapist time.	CBT	G	Web	7
Umanodan et al (2014) [25]	SMT program in employees: Self-paced program. (1) behavioral techniques, (2) communication techniques, and (3) cognitive techniques. Each part was divided into 2 topics on the basis of cognitive behavioral skills: (1) problem-solving and time management skills for the behavioral techniques section, (2) assertion and delegation skills for the communication techniques section, and (3) cognitive restructuring and causal attribution skills for the cognitive techniques section. 2-phased approach: skill acquisition phase and a practice phase. Weekly emails (for encouragement, congratulation, and application enhancement).	CBT	UG (R)	PC	7
Wiegand et al (2010) [38]	Comprehensive program for reducing stress: Group I: Daily use of olfactory care products plus an Internet-based program focusing on stress reduction, prevention and behavioral modification, (eg, muscle relaxation, relaxed breathing, and meditation). Periodic feedback reports are provided. Group II: Internet-based program only (no care products).	ALT, TWC	UG	Web	12

Study (Label)	Content	Type[a]	Guidance[b]	Delivery	Length (weeks)
Wolever et al (2012) [37]	Mindfulness at Work Intervention: 12 modules. Online virtual classroom with real-time bidirectional communication with an experienced mindfulness trainer (14 hours in total). The program teaches mindfulness practices that explicitly target work-related stress, work-life balance, and self-care. Includes brief exercises designed to be used at work.	TWC	G	Web	12
Yamagishi et al (2008) [57]	Web-based career identity training for stress management: 4 modules. (1) definition of career identity, (2): cognition of own career identity, (3): characteristics of nurses' career identity, (4): career goal management and planning.	ALT	UG	Web	3
Zetterqvist et al (2003) [26]	Internet-based self-help stress-management program: Each of the 6 modules consists of 3 sections: relaxation (progressive, conditioned, differential, cue controlled, applied), additional exercises (problem solving, time management and cognitive and behavioral responses to stress), and information (sleep management, eating habits, exercise, stress at work, positive activities, limit setting). Exercises were sent in and participants received feedback as a prompt to continue; includes reminders.	CBT	G	Web	6

[a] ALT: alternative; CBT: cognitive behavioral therapy; TWC: third-wave cognitive behavioral therapy.

[b] AFG: Adherence-focused guidance; G: guided; UG: unguided; UG (R): unguided with reminders via mail or telephone.

Source:

Heber E, Ebert DD, Lehr D, Cuijpers P, Berking M, Nobis S, Riper H. The Benefit of Web- and Computer-Based Interventions for Stress: A Systematic Review and Meta-Analysis. J Med Internet Res 2017;19(2):e32
